# *Akkermansia muciniphila* Ameliorates *Clostridioides difficile* Infection in Mice by Modulating the Intestinal Microbiome and Metabolites

**DOI:** 10.3389/fmicb.2022.841920

**Published:** 2022-05-18

**Authors:** Zhengjie Wu, Qiaomai Xu, Silan Gu, Yunbo Chen, Longxian Lv, Beiwen Zheng, Qiangqiang Wang, Kaicen Wang, Shuting Wang, Jiafeng Xia, Liya Yang, Xiaoyuan Bian, Xianwan Jiang, Lisi Zheng, Lanjuan Li

**Affiliations:** ^1^State Key Laboratory for Diagnosis and Treatment of Infectious Diseases, National Clinical Research Centre for Infectious Diseases, Collaborative Innovation Center for Diagnosis and Treatment of Infectious Diseases, The First Affiliated Hospital, Zhejiang University School of Medicine, Hangzhou, China; ^2^Bacterial Research Platform, Jinan Microecological Biomedicine Shandong Laboratory, Jinan, China

**Keywords:** *Clostridioides difficile*, probiotic, *Akkermansia muciniphila*, intestinal microbiota, cytokines

## Abstract

*Clostridioides difficile* is a common cause of nosocomial infection. Antibiotic-induced dysbiosis in the intestinal microbiota is a core cause of *C. difficile* infection (CDI). *Akkermansia muciniphila* plays an active role in maintaining gastrointestinal balance and might offer the protective effects on CDI as probiotics. Here, we investigated the effects and mechanisms of *A. muciniphila* on CDI. C57BL/6 mice (*n* = 29) were administered *A. muciniphila* Muc*^T^* (3 × 10^9^ CFUs, 0.2 mL) or phosphate-buffered saline (PBS) by oral gavage for 2 weeks. Mice were pretreated with an antibiotic cocktail and subsequently challenged with the *C. difficile* strain VPI 10463. *A. muciniphila* treatment prevented weight loss in mice and reduced the histological injury of the colon. And it also alleviated inflammation and improved the barrier function of the intestine. The administration effects of *A. muciniphila* may be associated with an increase in short-chain fatty acid production and the maintenance of bile acids’ steady-state. Our results provide evidence that administration of *A. muciniphila* to CDI mice, with an imbalance in the microbial community structure, lead to a decrease in abundance of members of the Enterobacteriaceae and Enterococcaceae. In short, *A. muciniphila* shows a potential anti-CDI role by modulating gut microbiota and the metabolome.

## Introduction

*Clostridioides difficile* is a Gram-positive and spore-forming anaerobic bacteria, which can cause diarrhea and severe complications such as pseudomembranous colitis (Gerding et al., 1995; Fletcher et al., 2021). The pathogenesis of *C. difficile* is mainly derived by two toxins, toxin A and toxin B, which induce intestinal inflammation and tissue damage (Kuehne et al., 2010). The indiscriminate use of antibiotics is currently regarded as the most common reason for gut microbiota imbalance. If this disruption reaches a certain level, the host can develop *C. difficile*-associated disease (CDAD) (Tang et al., 2016). In general, *C. difficile* infections (CDI) alter the gut microbiota, decreasing colonization resistance against *C. difficile*. Antibiotics such as MTZ, vancomycin, and fidaxomicin are still the main therapy for *C. difficile* (Mushtaq, 2018). In some cases, antibiotics even promote CDIs, which can take over after the symbiotic microflora is damaged by antibiotics (Theriot et al., 2014). These broad-spectrum antibiotics affect the healthy gut microbiota and hinder colonization resistance by reducing the abundance of intestinal microbes that resist *C. difficile* (van Werkhoven et al., 2021). In turn, the reduced microbiota diversity induces an elevated level of primary bile acids (BAs), which facilitate the germination of *C. difficile* spores and reduce the concentration of secondary BAs that inhibit the growth of *C. difficile* (de Aguiar Vallim et al., 2013). In addition to gut microbiota disorders, intestinal inflammation plays a complex role in CDI-mediated diseases (Shen, 2012). Though antibiotics are considered the most effective therapy in clinical practice, the risks of developing antibiotics resistance and further gut microbiota perturbation are important (McDonald et al., 2018b; Rao and Malani, 2020).

Further, the diversity of gut microbes is related to the recurrence of CDI and the severity of the disease. Fecal microbiota transplantation (FMT) can effectively treat recurrent CDI by recovering the gut microbiome (van Nood et al., 2013), but there are concerns regarding its long-term safety. An integrated gut barrier with a healthy microbiota is vital to resist *C. difficile* pathogenesis. Therefore, supplementing bacteria in a timely manner to establish a health-associated microbiome seems more reasonable. The regulation of the gut microbiome may be critical for preventing and treating CDI. Probiotics improve the balance of microorganisms that populate the gut and reduce CDI in high-risk patients receiving antibiotics (Maziade et al., 2015). Thus, effective alternative non-antibiotic therapies are urgently needed that will be helpful to design strategies to maintain the normal balance of gut microflora. Recently, the focus has shifted to bacteriotherapy which has gradually become an accepted alternative for CDI treatment (Lawley et al., 2012).

*Akkermansia muciniphila* – a mucophilic anaerobic bacteria, is one such bacterium representing 3–5% of the microbial community in healthy individuals (Belzer and de Vos, 2012; Becken et al., 2021). It has several phenotypes such as oxygen tolerance, adherence to epithelial cells, and bacterial aggregation. It is the first and representative member of the *Verrucomicrobia* found in the human gut (Derrien et al., 2010), whose metabolic byproducts, such as short-chain fatty acids (SCFAs), benefit other gut bacteria. Additionally, this bacterium is associated with the gut barrier, inflammation, and immune response (Reunanen et al., 2015). *A. muciniphila* stimulates mucin production (van der Lugt et al., 2019) and is associated with inflammatory bowel disease (Png et al., 2010), metabolic syndrome (Zhang et al., 2009; Santacruz et al., 2010), liver injury (Wu W. et al., 2017), and regulation of gut homeostasis (Derrien et al., 2010). Although *A. muciniphila* is a mucin-degrading bacterium, it can stimulate mucin production, increase intestinal mucous layer thickness and intestinal barrier integrity (Everard et al., 2013), protect the intestinal tract from pathogens through competitive rejection (van der Lugt et al., 2019). *A. muciniphila* and its membrane protein Amuc-1100 played an important probiotic role, is considered a very promising probiotic. However, the protective effects of *A. muciniphila* and its microbial and immunomodulatory properties have not been extensively studied. Therefore, the aim of this study was to explore the protective effects of *A. muciniphila* on CDI colitis and the potential underlying mechanisms.

## Materials and Methods

### Strains and Culture Conditions

*Akkermansia muciniphila* Muc*^T^* (= ATCC BAA-835*^T^* = CIP 107961*^T^*) was grown anaerobically in a BHI medium at 37°C for 48 h (Derrien et al., 2004). The broth was centrifuged at 8,000 *g* at 4°C for 10 min, washed, and re-suspended in sterile phosphate-buffered saline (PBS). We used a non-hyper virulent strain of *C. difficile* (strain VPI 10463) known to produce a high-level of toxin (Merrigan et al., 2010). The *C. difficile* VPI 10463 (ATCC 43255, binary toxin-negative, toxin A-positive, and toxin B-positive) was cultured anaerobically at 37°C for 24 h in anaerobic conditions in Difco cooked meat medium (BD Diagnostic Systems, United States). The culture was centrifuged (3,200 *g*, 10 min, 4°C). The sediments were washed twice with sterile PBS and re-suspended for further use (Chen et al., 2008).

### Mice and Experimental Design

**FIGURE 1:**
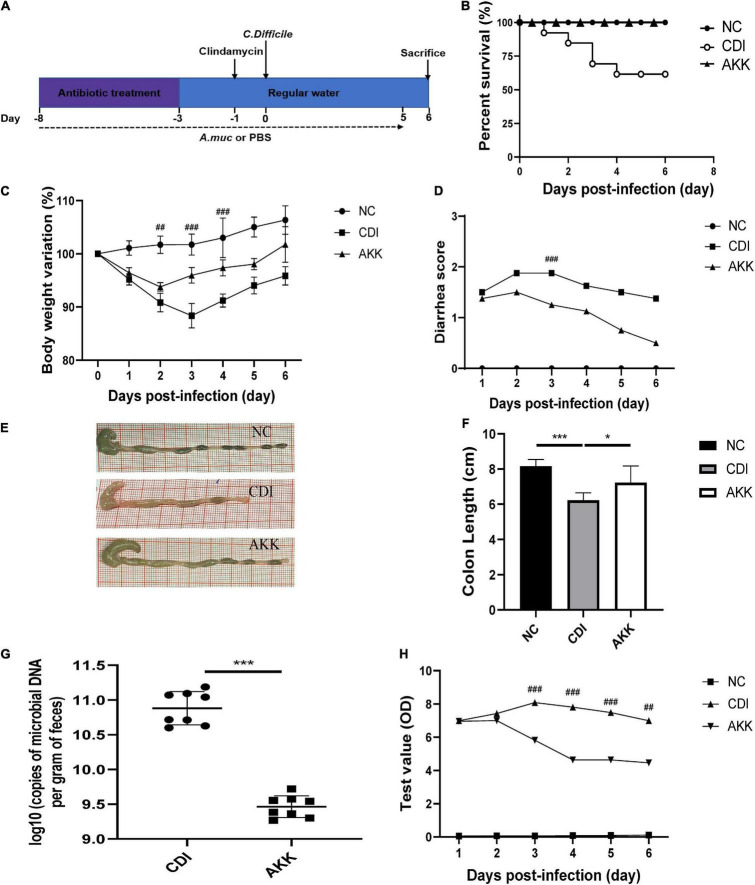
*Akkermansia muciniphila* administration reduced *C. difficile*-induced colon damage. **(A)** Overall schematic experimental plan. **(B)** Kaplan–Meier survival curve of NC, CDI, and AKK groups. **(C)** Body weight changes, **(D)** diarrhea scores during infection. **(E)** Representative colon pictures and **(F)** colon length after *A. muciniphila* administration. **(G)** *C. difficile* load in feces was detected on day 6. **(H)** Fecal toxins A and B were detected on post-infection days and expressed as test values (ODs). *P < 0.05, **P < 0.01, ***P < 0.001, ^##^*P* < 0.01,^ ###^*P* < 0.001 for CDI group vs. AKK group.

Female C57BL/6 mice (6–8 weeks old, SLAC Lab, Shanghai, China) were housed under controlled conditions (specific pathogen free). We used a uniform CDI mouse model (Chen et al., 2008). Briefly, mice were grouped randomly: 0.2 mL PBS [CDI group, *n* = 13; standard control [NC] group, *n* = 8] and 0.2 mL *A. muciniphila* suspension [3 × 10^9^ CFUs, *A. muciniphila* (AKK) group, *n* = 8] once daily by oral gavage from day-8 to day 5 (Figure 1A). For *C. difficile in vivo* infection, AKK group and CDI group received antibiotics cocktail (0.4 mg/mL kanamycin, 0.035 mg/mL gentamicin, 850 U/mL colistin, 0.215 mg/mL metronidazole, and 0.045 mg/mL vancomycin) in drinking water for 5 days followed by 2 days of normal water. Mice were intraperitoneally injected with clindamycin (10 mg/kg) 1 day before infection. On day 0, mice were given oral gavage of *C. difficile* (10^8^ CFUs, 0.2 mL). The diarrhea was graded as 0, normal stool; 1, loose stool; 2, liquid feces or soiled tail (Tam et al., 2018). According to the manufacturers’ instructions, toxins A and B were detected in feces using an enzyme immunoassay (VIDAS *C. difficile* Toxin A and B; bioMerieux SA, Marcy-l’Étoile, France). The experiment was approved by the Animal Experimental Ethical Inspection of The First Affiliated Hospital, Zhejiang University School of Medicine (Zhejiang, China) (1-7-2021).

### Histopathological Analysis, Immunohistochemical Staining, and Immunofluorescence

Upon euthanasia, distal colon segments were immediately postfixed in 10% formalin. The tissue sample was embedded in paraffin and stained with hematoxylin and eosin. The degree of enteritis, which uses a scoring system as previously described (Chen et al., 2008), consists of epithelial cell damage (score: 0–3), congestion/edema (score: 0–3), and neutrophil infiltration (score: 0–3). Two independent pathology professors double-blindly evaluated these sections. Alcian blue and periodic acid-Schiff (AB-PAS) staining were performed with the instructions (Solarbio). The sections were stained with p-mTOR, beclin1, zonula occludens-1 (ZO-1), occludin, and claudin-1 antibodies as previously described (Chung et al., 2014). Images were managed using the Zeiss LSM T-PMT confocal microscope (Zeiss, Jena, Germany).

### Transmission Electron Microscopy

The proximal colon samples were collected and immediately transferred to glutaraldehyde (2.5%) at 4°C for 4 h, postfixed with 1% OsO_4_ and dehydrated in graded alcohol concentrations, and embedded in Spurr resin (SPI-CHEM). The specimen was sectioned using the LEICA EM UC7 ultratome (Leica Microsystems GmbH, Wetzlar, Germany), and sections were stained with uranyl acetate (SPI-CHEM) and alkaline lead citrate. These sections were observed by transmission electron microscopy (TEM) (H-7650; Hitachi, Tokyo, Japan).

### Serum and Colon Parameter Analyses

The limulus amebocyte lysate (LAL) assay detected and quantitated the endotoxins. Serum LAL levels were measured using the LAL Chromogenic Endpoint Assay (Hycult Biotechnology, Uden, Netherlands). Lipopolysaccharide (LPS)-binding protein (LBP) concentrations were detected using an enzyme-linked immunosorbent assay (ELISA) kit (Abcam, Cambridge, MA, United States). The serum and colon cytokines were measured with the Bio-Plex Pro Mouse Cytokine 23-Plex Assay Kit and Chemokine 31-Plex Assay Kit (Bio-Rad, Hercules, CA, United States). The 23-plex contains granulocyte colony-stimulating factor (G-CSF), monocyte chemoattractant protein-1 (MCP-1), interleukin 17A (IL-17A), IL-1α, IL-6, and tumor necrosis factor-alpha (TNF-α). The 31-plex contains: IL-1β, IL-6, IP-10/C-X-C motif chemokine ligand 10 (CXCL10), MCP-1/CCL2, MDC/CCL22, BCA-1/CXCL13, I-309/C-C motif chemokine ligand 1 (CCL1), MIP-1α/CCL3, MIP-1β/CCL4, RANTES/CCL5, TARC/CCL17, and TNF-α.

### Quantitative Real-Time PCR

Total RNA of colorectal tissue was reverse transcribed into cDNA using the RNeasy Plus Mini Kit (Qiagen, Valencia, CA, United States) and PrimeScript RT Master Kit (Takara Biomedicals, Kusatsu, Japan). The mRNA relative abundance was measured in triplicate using the Premix Ex Taq (Takara Biomedicals) using the VIIA7 Real-time PCR System (Applied Biosystems, Beverly, MA, United States). The relative mRNA expression was determined using the 2^–ΔΔ*CT*^ method and normalized to the internal control β-actin. The primer sequences are listed in Supplementary Table 1.

### Stool DNA Extraction, *Clostridioides difficile*, and *Akkermansia muciniphila* Quantification

Fecal samples and extracted DNA through the DNeasy PowerSoil Pro Kit (Qiagen). DNA from fecal samples was used in quantitative PCR. Previously reported primers were used to detect and quantify *C. difficile* (Pereira et al., 2020) and *A. muciniphila* (Everard et al., 2013). The standard curve used serial dilutions of isolating DNA to quantify the DNA. The samples were tested in duplicate.

### Fecal Microbiome Analysis

DNA from fecal samples on day 6 was extracted by the DNeasy PowerSoil Pro Kit (Qiagen) (NC = 8, CDI = 8, AKK = 8). The 16S rRNA was amplified using primer pairs 338F (5′-ACTCCTACGGGAGGCAGCAG-3′) and 806R (5′-GGACTACHVGGGTWTCTAAT-3′) corresponding to the V3–V4 region. Sequencing was performed using the Illumina MiSeq PE300 system and was analyzed on Majorbio Cloud Platform^1^ as previously described (Gu et al., 2020). Briefly, operational taxonomic units (OTUs) were clustered using UPARSE version 7.1 (Edgar, 2013) with a 0.97 threshold. Representative reads of each OTU were selected, and taxonomic data were then assigned using the RDP classifier and display a 70% confidence threshold. Alpha diversity and beta diversity were calculated using QIIME software. Alpha diversity was estimated with the Chao 1 index and Shannon index. Using the Adonis function, we performed a permutational multivariate analysis of variance (PERMANOVA) based on Bray–Curtis distances. Beta diversity was assessed by the Bray–Curtis distance and presented by principal coordinate analysis (PCoA). The differences between groups in taxonomic composition taxa were analyzed using the linear discriminant analysis (LDA) effect size (LEfSe) analysis, and LDA scores greater >4 were defined as discriminative taxa. The sequencing data has been uploaded to the NCBI Sequence Read Archive (SRA) database (PRJNA 766049).

### Fecal Bile Acid Composition Analysis

Bile acids (TCA, taurocholic acid; βMCA, β-muricholic acid; CA, cholic acid; αMCA, α-muricholic acid; UDCA, ursodeoxycholic acid; CDCA, chenodeoxycholic acid; DCA, deoxycholic acid; ωMCA, ω-muricholic acid; HDCA, hyodeoxycholic acid; muroCA, murocholic acid; βDCA, β-deoxycholic acid; and LCA, lithocholic acid) in colon contents were detected according to previously described protocol (Xie et al., 2015; Yang et al., 2021) and purchased from Steraloids, Inc. (Newport, RI, United States). Briefly, colon contents were mixed with 200 μL acetonitrile/methanol (volume 8:2), which contained internal standards (GCA-d4, TCA-d4, GDCA-d4, CA-d4, DCA-d4, and LCA-d4). After homogenization and centrifugation (13,500 rpm, 20 min, 4°C), 10 μL supernatant was diluted with 90 μL acetonitrile/methanol (80/20) and ultrapure water mixture solution (volume 1:1). Colon contents BAs were detected by ultra-performance liquid chromatography-tandem mass spectrometry (UPLC-MS/MS) (ACQUITY UPLC-Xevo TQ-S, Waters Corp., Milford, MA, United States).

### Short-Chain Fatty Acid Quantification

Short-chain fatty acids in feces were detected according to the previous method (Bian et al., 2020). Stool samples were mixed with 500 μl water (hexanoic acid-d3, internal standard, 10 μg/ml). After homogenization, samples were centrifugated at 15,000 rpm and 4°C for 5 min and mixed the supernatant with the equivalent volume mixture of ethyl acetate/sulfuric acid (v/v = 10:1). After centrifugation and derivatization, the supernatant was run and analyzed by GC/MS (Agilent Technologies, Santa Clara, CA, United States).

### Statistical Analyses

GraphPad Prism (GraphPad Software, Inc., CA, United States) and SPSS 20.0 (SPSS, Inc., Chicago, IL, United States) was used for statistical analyses. For statistical significance, either one-way ANOVA followed by the Tukey’s test or Kruskal–Wallis test was used. Kaplan–Meier survival curves used the log-rank (Mantel–Cox) test. Spearman’s rank correlation test analyzed correlations between relevant variables. *P* < 0.05 was considered statistically significant.

## Results

### *Akkermansia muciniphila* Treatment Improves the *Clostridioides difficile* Infection-Induced Clinical Outcome in Mice

As previously reported, body weight loss during CDI-induced infection (Xu et al., 2018) can be a marker of disease progression. Therefore, we used weight loss during the disease course to measure CDI disease severity. The CDI group showed distinct weight loss after infection (D3, CDI group vs. NC group, *P* < 0.001; Figure 1C), and five mice died by day 4 (Figure 1B; *P* < 0.05). All mice in the NC group and AKK group survived. Oral supplementation of *A. muciniphila* reduced body weight loss after infection (Figure 1C), alleviated the diarrhea score (Figure 1D), relieved colon shortening (NC vs. CDI groups, *P* < 0.001; AKK group vs. NC group, *P* < 0.05; AKK group vs. CDI group, *P* < 0.05; Figures 1E,F). Post-infection fecal samples showed a reduction of *C. difficile* burden and its toxins with AKK treatment (*P* < 0.001; Figures 1G,H). Therefore, probiotics significantly reduced CDI-induced clinical symptoms. To establish the mechanism of protection induced by *A. muciniphila* in CDI- induced mice we studied histopathological analysis and immune response to treat such infection.

### *Akkermansia muciniphila* Improves the Intestinal Barrier Under *Clostridioides difficile*-Induced Colon Injury

**FIGURE 2:**
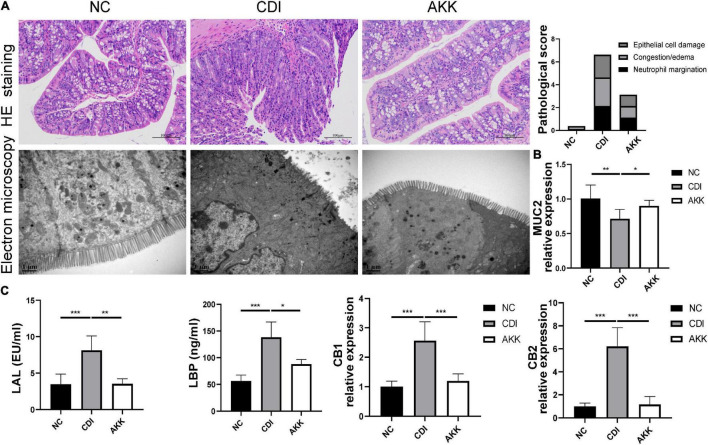
*Akkermansia muciniphila* reduced colon epithelial injury. **(A)** Representative images of hematoxylin and eosin staining and ultrastructure under TEM (left panel). Histopathology scores among three groups (right panel). **(B)** Colon mRNA expression of MUC2. **(C)** Serum LAL and LBP levels and colon mRNA expression of CB1 and CB2. *P < 0.05, **P < 0.01, ***P < 0.001.

Immunohistological staining suggested that *A. muciniphila* reduced histological injury in the distal colon, such as destroying the histological structure and epithelial barrier (Figure 2A and Supplementary Figure 1). TEM showed that intestinal epithelial cell (IEC) microvilli were ruptured in the CDI group. This disruption across the brush border was reduced in the AKK group (Figure 2A). We speculated that *A. muciniphila* may play a role by enhancing the mucosal barrier. Immunofluorescence staining and quantitative real-time PCR (qPCR) were used to assess the expression of tight junction proteins (Tjps) and mRNA, respectively. As shown in Supplementary Figure 1A, the AKK group showed stable mucosal integrity of the colon tissue and increased fluorescence intensity of the ZO-1, occludin, and claudin-1 compared to the CDI group. The mRNA expression of Tjps in the colonic tissue of the AKK group was higher than that of the CDI group (occludin, ZO-1/Tjps, claudin-1, *P* < 0.05 for all; Supplementary Figure 1A). Bacteria and their metabolites, such as LPS, invade the intestines and blood when a leaky gut occurs. LBP is a carrier for LPS and helps its macrophages recognize. ELISA measured serum LBP and LAL to assess the severity of bacterial translocation and inflammation. The results showed that serum LBP and LAL levels in the CDI group were higher than those in the NC and AKK groups (*P* < 0.05; Figure 2C). In addition, intestinal permeability was evaluated by assessing MUC2 and cannabinoid receptor (CB) levels. CB1 and CB2 had significantly increased expression in the CDI group than the NC group (*P* < 0.05), meanwhile the expression was downregulated in the AKK group than the CDI group (*P* < 0.05; Figures 2B,C). We also examined the mucin layer covering the proximal colon by AB-PAS staining in Supplementary Figure 1B. CDI group damaged the mucous layer, while *A. muciniphila* protected the mucous layer. As noted above, *C. difficile* caused severe intestinal mucosal injury, while probiotics treatment improved symptoms.

### Effects of *Akkermansia muciniphila* Treatment on the Local and Systemic Immune Response

**FIGURE 3:**
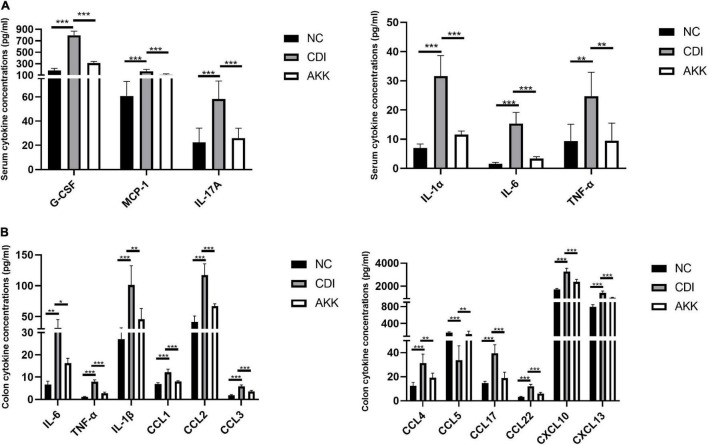
*Akkermansia muciniphila* ameliorated local and systemic anti-inflammation. **(A)** Serum concentrations of G-CSF, MCP-1, IL-17A, IL-1α, IL-6, and TNF-α. **(B)** Colon tissue IL-6, TNF-α, IL-1β, CCL1, CCL2, CCL3, CCL4, CCL5, CCL17, CCL22, CXCL10, and CXCL13. *P < 0.05, **P < 0.01, ***P < 0.001.

Inflammatory cytokines are related to the pathogenesis mechanism of CDI colitis. We measured serum and colon tissue cytokine levels to explore the protective effects of *A. muciniphila* on intestinal inflammation. Infected mice were detected with a series of elevated inflammatory cytokines in previous study (Pawlowski et al., 2010), increased levels of serum cytokines G-CSF, MCP-1, IL-17A, IL-1α, IL-6, and TNF-α were observed in CDI colitis mice. While the administration of *A. muciniphila* significantly reduced the levels of these cytokines (*P* < 0.05; Figure 3A). In contrast with the NC group, IL-6, TNF-α, IL-1β, CCL1, CCL2, CCL3, CCL4, CCL5, CCL17, CCL22, CXCL10, and CXCL13 were elevated significantly in CDI mice, and are related to intestinal inflammation (*P* < 0.05; Figure 3B). These cytokine concentrations were downregulated considerably after the administration of *A. muciniphila* (*P* < 0.05; Figure 3B). Thus, probiotics reduce inflammation levels and exhibit anti-inflammatory properties.

### *Akkermansia muciniphila* Mediates *Clostridioides difficile* Altered Autophagy and Innate Immunity in the Colon

**FIGURE 4:**
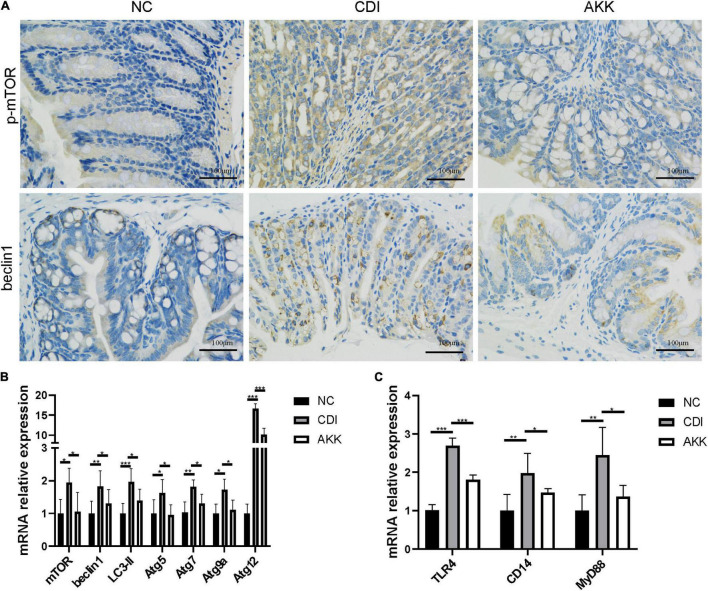
*Akkermansia muciniphila* relieved CDI-induced inflammation. **(A)** Representative immunofluorescence staining of p-mTOR and beclin1 in the colon. **(B)** Relative colon mRNA expression of mTOR, beclin1, LC3-II, Atg5, Atg7, Atg9a, and Atg12 in the colon. **(C)** Relative mRNA expression of TLR4, CD14, and MyD88 in the colon. *P < 0.05, **P < 0.01, ***P < 0.001.

Due to the mechanism of toxins A/B-induced IEC injury, we investigated the mammalian target of rapamycin (mTOR) signaling pathway to explore intestinal inflammation in CDI mice. The treatment decreased the autophagy-related protein expression (Figure 4A) and mRNA level (Figure 4B). There was reduced expression of phosphorylated mTOR (p-mTOR) and beclin1 in the colon epithelial cells (Figure 4A). The gene expression of autophagy-related genes such as mTOR, beclin1, light chain 3 (LC3)-II, autophagy-related gene 5 (Atg5), Atg7, Atg9a, and Atg12 were more elevated in the CDI group than in the AKK group (*P* < 0.05; Figure 4B). Similarly, the mRNA expression of immune markers such as Toll-like receptor 4 (TLR4), cluster of differentiation 14 (CD14, a co-receptor of TLR4), and myeloid differentiation 88 (MyD88) were downregulated after *A. muciniphila* administration (*P* < 0.001, *P* < 0.05, and *P* < 0.05, respectively; Figure 4C).

### *Akkermansia muciniphila* Alleviates *Clostridioides difficile* Infection-Induced Microbiome Dysbiosis

**FIGURE 5:**
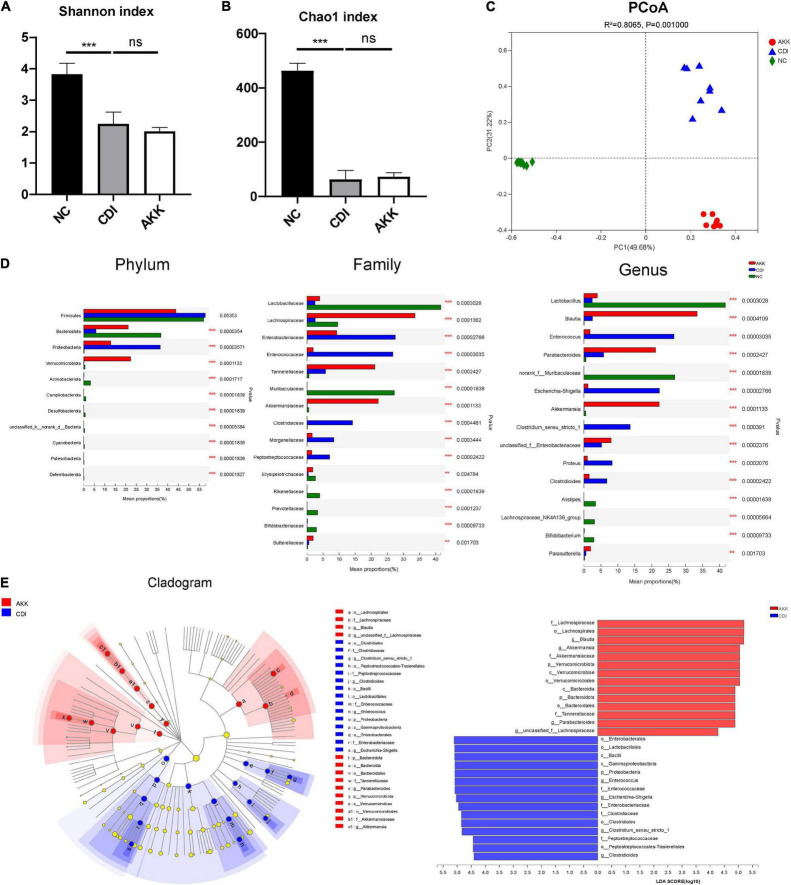
Effects of pretreatment with *A. muciniphila* on the changes of gut microbial community structure. **(A,B)** The Shannon index and Chao1 index evaluated alpha diversity. **(C)** The PCoA plot by Bray–Curtis analysis. **(D)** Comparison of taxa at the phylum, family, and genus levels among three groups. **(E)** LEfSe analyses between the CDI group (blue) and the AKK group (red) (LDA score >4). ***P < 0.001.

To study the microbiome composition, we used 16S rRNA gene sequencing and obtained a total of 1,132,433 sequences (47,184 reads per sample). The CDI group showed a decline in microbiome diversity (Shannon index, Figure 5A) and richness (Chao1 index, Figure 5B). Furthermore, the PCoA plot showed significantly different microbiota among the three groups (Figure 5C, PERMANOVA, *R*^2^ = 0.8065, p_adjust = 0.001). These results suggested the reduced abundance of *A. muciniphila* in the CDI group (Figure 5D), with a concomitant increased load of *A. muciniphila* in the AKK group (Supplementary Figure 2A).

The relative taxon abundance at the phylum level showed that the CDI group was enriched with *Proteobacteria* and depleted of Bacteroidota (Figure 5D). While at the family level, the CDI group considerably lacked Lactobacillaceae, Lachnospiraceae, and Akkermansiaceae but was enriched in Enterobacteriaceae, Enterococcaceae, Clostridiaceae, Morganellaceae, and Peptostreptococcaceae. The oral administration of *A. muciniphila* significantly improved this enrichment and depletion.

Linear discriminant analysis effect size analysis was used to identify taxa, and the most diverse results (LDA score >4) were analyzed. Compared with the NC group, the phylum Bacteroidota, class Bacteroidia, order Bacteroidales, and families Rikenellaceae, Muribaculaceae, and Prevotellaceae in the CDI group were depleted (Supplementary Figure 2B). Meanwhile, the phylum Actinobacteriota and the affiliated class Actinobacteria, order Bifidobacteriales, family Bifidobacteriaceae, and genus *Bifidobacterium* were also depleted in the CDI group. At the family level, Lachnospiraceae, Lactobacillaceae, and Erysipelotrichaceae in the CDI group were also reduced compared with the NC group. At the same time, the enrichment of families Peptostreptococcaceae, Enterococcaceae, Enterobacteriaceae, and Morganellaceae in the CDI group led to an imbalance of intestinal flora.

Compared with the CDI group, the AKK group showed depletion of opportunistic pathogens in the *Enterococcus* (belonging to family Enterococcaceae and order Lactobacillales), *Escherichia–Shigella* (belonging to family Enterobacteriaceae, order Enterobacterales, class Gammaproteobacteria and phylum Proteobacteria), *Clostridioides*, and *Clostridium_sensu_stricto_1* (Figure 5E). In addition, the AKK group was enriched for the genera *Blautia* (belonging to family Lachnospiraceae and order Lachnospirales), *Parabacteroides* (belonging to family Tannerellaceae, order Bacteroidales, class Bacteroidia, and phylum Bacteroidota), and *Akkermansia* compared with the CDI group, showing the role of *A. muciniphila* in restoring gut microbiota. Next, we studied the biochemical mechanisms associated with CDI’s *A. muciniphila* mediated protection.

### *Akkermansia muciniphila* Improves Bile Acid and Short-Chain Fatty Acid Metabolism

**FIGURE 6:**
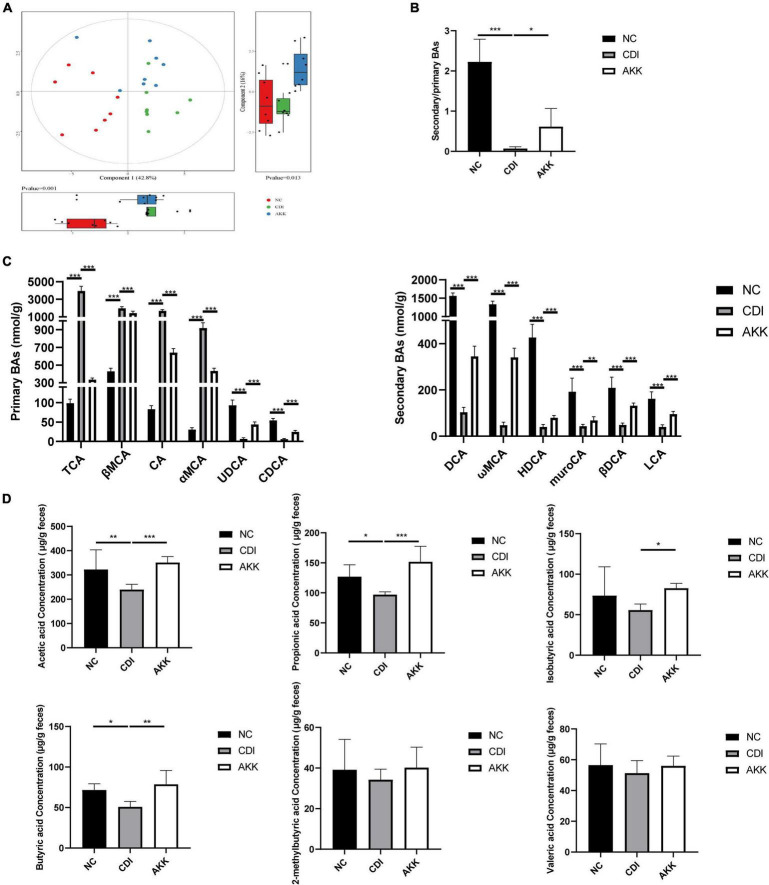
Effects of *A. muciniphila* on BA and SCFA metabolism. **(A)** PLS-DA score plot comparing the BA metabolite profile of three groups. **(B)** The ratio of secondary BAs to primary BAs. **(C)** Primary BA (left) and secondary BA (right) concentrations. **(D)** The fecal SCFA concentrations were measured using GC/MS including acetic acid, propionic acid, isobutyric acid, butyric acid,2-methylbutyric acid, and valeric acid. *P < 0.05, **P < 0.01, ***P < 0.001.

We detected the BA levels in the colon by UPLC-MS/MS. Partial least squares-discriminant analysis (PLS-DA) showed distinct clustering of the BAs among the three groups (Figure 6A), which showed alteration of BA metabolism by CDI. The CDI group showed decreased secondary BAs, DCA, ωMCA, HDCA, muroCA, βDCA, LCA, and UDCA (Figure 6C). Compared to the NC group, the levels of primary BAs were significantly raised in the CDI group. However, the level of secondary BA was restored, and primary BAs such as CDCA and CA were decreased after *A. muciniphila* administration (Figure 6C). The AKK group had an increased secondary BA to primary BA ratio than the CDI group (Figure 6B).

As shown in Figure 6D, we determined six major SCFAs in the fecal samples. The AKK group showed a significant increase in SCFA production. The acetic acid, propionic acid, and butyric acid concentrations were significantly decreased in the CDI group (CDI vs. NC: *P* < 0.05; Figure 6D). Furthermore, *A. muciniphila* administration increased the concentrations of acetic acid, propionic acid, isobutyric acid, and butyric acid (CDI vs. AKK: *P* < 0.05; Figure 6D). Thus, probiotics alter metabolic disorders caused by disease.

### Altered Gut Microbiota, Fecal Metabolome, and Immunity Indexes Are Correlated With Each Other in *Clostridioides difficile* Infection

**FIGURE 7:**
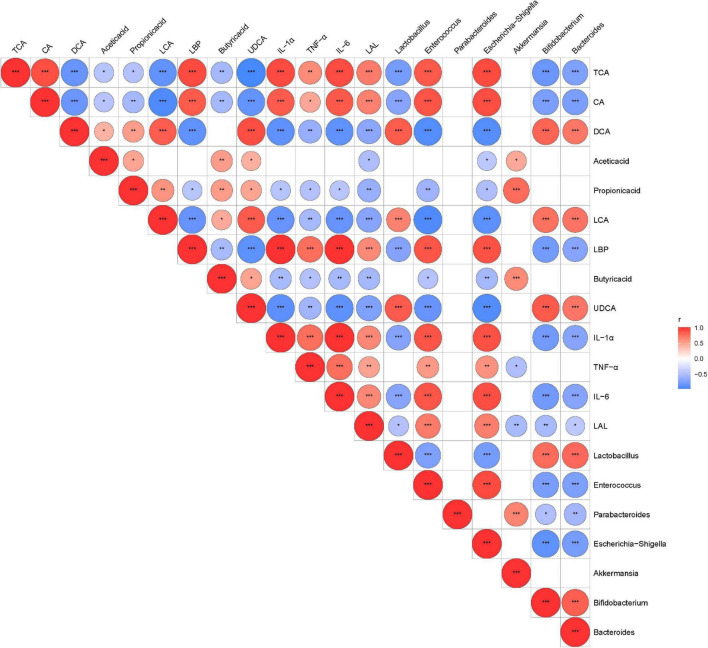
The relationship among the altered intestinal microbiota, metabolites, and inflammatory indexes were evaluated by Spearman’s correlation analysis. Red represents a positive correlation; blue represents a negative correlation. Red and blue represent positive and negative correlation, respectively. *P < 0.05, **P < 0.01, ***P < 0.001.

We performed Spearman’s correlation analysis between discriminative bacteria abundance and metabolic and serum inflammatory indexes to explore the relationship between the changed gut microbiota structures and disease severity. Our results showed that the relative abundance of *A. muciniphila*-modified bacteria was related to inflammatory indexes and mucosal barrier indicators (Figure 7). Potentially beneficial bacteria such as *Bifidobacterium*, *Bacteroides*, and *Lactobacillus* were negatively correlated with cytokines (e.g., IL-1α, IL-6, and TNF-α) but were positively correlated with SCFAs and secondary BAs. Opportunistic pathogens such as *Enterococcus* and *Escherichia–Shigella* were negatively correlated with SCFAs and secondary BAs but positively correlated with inflammatory indexes and primary BAs.

## Discussion

*Clostridioides difficile* treatment involves using antibiotics against the pathogen, thereby reducing toxin levels and damage to the gut epithelium. Antibiotic treatment does not promote the recovery of the microbiome of CDI patients and may further destroy the local microbiota and cause disease recurrence. This study found that *A. muciniphila* can reduce CDI mice mortality, significantly ameliorating CD-induced systemic inflammation and the damaged intestinal barrier. Our study further suggests that the potential mechanism might regulate the intestinal microbiota and related microbial metabolism.

Some studies show that CDI patients and mouse model have a low abundance of *A. muciniphila* (Rodriguez et al., 2016; Deng et al., 2018; Pellissery et al., 2021), while an increased number of *A. muciniphila* was observed in other studies (Sangster et al., 2016; Vakili et al., 2020; Shoaei et al., 2021). This contradictory conclusion associates to various factors. The probiotic/harmful properties of bacteria are strain and dose-specific (Myers et al., 2006; Wu G. et al., 2017). Since the human intestine may be colonized by different *Akkermansia*-like spp. with specific phenotypes (Becken et al., 2021), recently, 16S rRNA sequences of a set of *A. muciniphila* strains isolated from humans and mice in China suggested that 97% sequence identity with that of the type strain *A. muciniphila* Muc*^T^* isolated from Europe (Guo et al., 2017). In addition to *A. muciniphila*, only one other *Akkermansia*-like spp. *Akkermansia glycaniphila* have been described (Ouwerkerk et al., 2016). Moreover, the average nucleotide identity between the *A. glycaniphila* and the *A. muciniphila* Muc*^T^* genome was found to be 79.7%. The genome of *A. muciniphila* Muc*^T^* involves one circular chromosome of 2.66 Mbp, and shared genes (29%) with its closest relatives in the *Verrucomicrobia* (van Passel et al., 2011). Most current studies on *A. muciniphila* focus on *A. muciniphila* Muc*^T^*. Therefore, we have used a standard *A. muciniphila* Muc*^T^* strain which can degrade and mucin and a swell known model for studying the probiotic microbiome in human gastrointestinal tract (Geerlings et al., 2018). Our study demonstrated that the administration of *A. muciniphila* can improve intestinal inflammation through multiple host regulation, such as microbial-host interaction and SCFAs. At the same time, these conflicting controversies also prompt us to further explore its therapeutic value with better forms and dosages of administration.

Through maintaining the intestinal microbiota and microbiota-derived metabolites, *A. muciniphila* regulates the immune response in the intestine. The pathophysiology of CDI is closely related to the production of toxins A and B, which are the two main pathogenic enterotoxins and cytotoxins, which mainly target intestinal IECs and cause pseudomembranous colitis (Kuehne et al., 2010). Our results showed that *A. muciniphila* reduced *C. difficile* bacterial burden and the production of toxins in feces. The destruction of colonic epithelium and inflammatory activities increases gut bacterial translocation, causing the produce of various cytokines and promoting inflammatory cell migration to the damaged sites. The inflammatory cytokines and genes related to epithelial integrity showed the destruction of the intestinal mucosal barrier in our study. However, in HE staining and immunofluorescence staining, intestine damage was not evident between the CDI and the NC groups. The probable cause is the location of pathological samples was limited, but several parameters, such as inflammatory markers, suggest *C. difficile* damage to the gut barrier. Our study showed a significant decrease in inflammatory markers associated with the immune response and protection against infection in the AKK group. The CDI group showed elevated serum levels of LBP and LAL. Meanwhile, the serum levels of cytokines G-CSF, MCP-1, IL-17A, IL-1α, IL-6, and TNF-α were considerably increased, with significant elevation in cytokines and chemokines IL-6, TNF-α, IL-1β, CCL1, CCL2, CCL3, CCL4, CCL17, CCL22, CXCL10, and CXCL13 in the colon tissue. The accumulation of LPS (TLR4 agonist) in the blood triggers an inflammatory response through the TLR4 pathway.

*Clostridioides difficile* Infection-induced intestinal inflammation is associated with the regulation of the gut microbiota and the activation of TLR4/NF-κB signaling (Kim et al., 2006). Our results showed that *C. difficile* led to a decrease in intestinal cell turnover, reduced number of goblet cells, decreased expression of mucin (MUC2) and Tjps (ZO-1, occludin, and claudin-1), and damage to the integrity of the intestinal mucosa. Meanwhile, activation of the NF-κB pathway usually induces autophagy (Criollo et al., 2012). Toxins induce the necrosis and apoptosis of IECs (Mahida et al., 1996). In this context, our results found that *C. difficile* significantly upregulated autophagy-related markers, thereby showing increased autophagy. Dysfunctional autophagy may induce chronic intestinal inflammation, leading to severe intestinal damage (Randall-Demllo et al., 2013). *A. muciniphila* may reduce inflammation by inhibiting the TLR4/MyD88 and mTOR signaling pathways in the intestine and protecting the mucosal immune barrier. We found that the administration of *A. muciniphila* was associated with reduced pro-inflammatory production in mouse serum and intestinal tissues to alleviate intestinal inflammation. In addition, *A. muciniphila* improved the morphological architecture of the colon tissue in the CDI group, increased the expression of Tjps and MUC2, and concomitantly decreased the CB1 and CB2 levels in the colonic mucosa. These data show that *A. muciniphila* may reverse the intestinal mucosal damage caused by CDI by enhancing cell-cell interactions and induce a promoting effect on the gut mucosal barrier (Everard et al., 2013).

Intestinal epithelial cells receive signals from potential pathogens or symbiotic microorganisms and respond to the altered intestine by regulating mucosal barriers and transmitting signaling to IECs (Xu et al., 2021). Bacterial metabolites signal intestinal microbes to immune cells in the intestinal lamina propria, such as SCFAs and BAs. SCFAs, especially acetic acid (produced by multiple bacterial groups like *A. muciniphila*), propionic acid (produced by some species of *Bacteroidetes* and *Firmicutes*), and butyric acid (produced by a small number of *Clostridium*), are the final products of intestinal bacterial fiber fermentation (Koh et al., 2016; Louis and Flint, 2017). They affect host physiology, including an energy source for colon cells, regulating the intestinal barrier, and affecting inflammatory responses (Correa-Oliveira et al., 2016). SCFAs also inhibit *C. difficile* toxin production (Yuille et al., 2020), reducing CDI (McDonald et al., 2018a; Fachi et al., 2020) and inhibiting spore growth (Battaglioli et al., 2018; Hryckowian et al., 2018; Neumann-Schaal et al., 2019). Like previous studies (McDonald et al., 2018a), the CDI group showed decreased butyric acid, propionic acid, acetic acid, and *A. muciniphila* restored SCFA metabolism, possibly by restoring the intestinal flora balance. *A. muciniphila* acts as an SCFA producer and can enhance barrier immunity (Bian et al., 2019).

The intestinal homeostasis exhibits colonization resistance to *C. difficile*, and gut microbial-derived secondary BAs exert functions (Thanissery et al., 2017). Some primary BAs, such as taurocholate, promote the spore germination of *C. difficile* (Sorg and Sonenshein, 2008). DCA, as well as LCA (Buffie et al., 2015), ω-MCA (Francis et al., 2013), and UDCA (Weingarden et al., 2016), inhibit vegetative cell growth and spore outgrowth. Secondary BAs show a positive association with *Akkermansia* levels (Pierre et al., 2016). Several studies have shown that an antibiotic-induced microbiota imbalance reduces the pool of secondary BAs (Zarrinpar et al., 2018). Similarly, other studies have suggested that the loss of secondary BAs reduces the activation of anti-inflammatory markers such as TGR5 and FXR, which may impair the inflammatory pathways and intestinal barrier function (Sinha et al., 2020). In our studies, primary BAs in CDI mice increased, while secondary BAs such as CA and CDCA decreased. *A. muciniphila* helps to restore the levels of secondary BAs.

Microbiome regulation has been proposed as a possible mechanism for *A. muciniphila* to prevent CDI. The complex and stable microbial community in the intestine is a natural barrier against *C. difficile*. The signs of decreased gut microbial diversity are reduced microbial colonization resistance and increased susceptibility to *C. difficile* (Chang et al., 2008). The inflammation caused by CDI will, in turn, lead to the loss of microbiota diversity (Theriot et al., 2014). In agreement with the previous report, CDI patients or animal models showed intestinal flora disorders, including decreased relative abundance of Bacteroidota and increased proportion of LPS-producing *Proteobacteria* in the intestinal flora. The AKK group showed a lower abundance of harmful bacteria *Enterococcus* and *Escherichia–Shigella*, which may relate to the regulation of gut microbiota. *Escherichia–Shigella* (belonging to phylum Proteobacteria) is an adherent-invasive bacteria and may aggravate colon inflammation. *Proteobacteria* is the primary source of intestinal endotoxin LPS. Its abundance depended on intestinal LPS (Lin et al., 2020). *Proteobacteria* cause inflammation (Shin et al., 2015) and provide an environment conducive to invading pathogens like *C. difficile* (Fuentes et al., 2014). Endotoxemia is relevant to the abundance of gut bacteria associated with LPS production. Our results showed these opportunistic pathogens were positively correlated with serum inflammatory indexes, suggesting their harmful role in gut microbiota.

Meanwhile, *A. muciniphila* significantly improved the abundance of beneficial bacteria such as *Blautia*, *Parabacteroides*, and *Akkermansia*, which alleviated intestinal inflammation in the CDI by ameliorating the intestinal barrier function and microbiota structure. These strains are acetate or butyrate producers, also can inhibit *C. difficile in vitro* coculture experiments (Ghimire et al., 2020). These strains used mannitol, sorbitol, or succinate as carbon sources, the nutrients used by *C. difficile* to invade and cause inflammation in the intestine (Theriot et al., 2014; Spiga et al., 2017).

One limitation of our study is that we only used female mice for CDI model as suggested in previous studies (Chen et al., 2008; Buffie et al., 2015). Though several studies have indicated that male and female mice showed clinical manifestations (Chen et al., 2008), performing equivalent studies in males will be important, and further experimental studies would further refine the issue.

In summary, *A. muciniphila* treatment ameliorated CDI by modulating intestine barrier function and maintaining the steady state of intestine microbiota, immunological function, and metabolite profiles. Our work puts forth a critical role of *A. muciniphila* as a sentinel of intestinal homeostasis during CDI. Therefore, *A. muciniphila* may be a promising therapeutic target for *C. difficile*-related colitis via modulation of the immune response. Further work should be directed toward understanding the precise molecular mechanisms underlying the protective function of *A. muciniphila* in microbe-microbe interactions.

## Conclusion

*Clostridioides difficile* is a common cause of hospital infection following antibiotic treatment, inducing a heavy burden on most hospitals. *A. muciniphila* has showed its active role in maintaining gastrointestinal balance and might prevent and control CDI as probiotics. Our study found that *A. muciniphila* can reduce CDI mice mortality, significantly ameliorating *C. difficile*-induced systemic inflammation and the damaged intestinal barrier.

## Data Availability Statement

The datasets presented in this study can be found in online repositories. The names of the repository/repositories and accession number(s) can be found below: https://www.ncbi.nlm.nih.gov/, PRJNA 766049.

## Ethics Statement

The animal study was reviewed and approved by the Animal Experimental Ethical Inspection of The First Affiliated Hospital, Zhejiang University School of Medicine (Zhejiang, China).

## Author Contributions

LJL, ZW, QX, and SG designed the experiments. ZW and QX analyzed the data. ZW wrote the manuscript. All authors reviewed the manuscript.

## Conflict of Interest

The authors declare that the research was conducted in the absence of any commercial or financial relationships that could be construed as a potential conflict of interest.

## Publisher’s Note

All claims expressed in this article are solely those of the authors and do not necessarily represent those of their affiliated organizations, or those of the publisher, the editors and the reviewers. Any product that may be evaluated in this article, or claim that may be made by its manufacturer, is not guaranteed or endorsed by the publisher.

## Footnotes

1
www.majorbio.com
