# Supplementary material for: Akkermansia muciniphila Ameliorates Clostridioides difficile Infection in Mice by Modulating the Intestinal Microbiome and Metabolites
Source: Front Microbiol. 2022 May 18;13:841920. doi: 10.3389/fmicb.2022.841920 (PMC9159907; doi:10.3389/fmicb.2022.841920)
Supplement: Supplementary file 1 [file Table_1.DOCX]

Supplementary Material

# Supplementary Figures and Tables

For more information on Supplementary Material and for details on the different file types accepted, please see [here](http://home.frontiersin.org/about/author-guidelines#SupplementaryMaterial).

1. **Supplementary Methods**

**1.1 Immunohistochemical experimental procedure**

Paraffin-embedded samples were cut into 4 µm thick sections and prepared in citric acid (pH 6.0; China National Pharmaceutical Group Chemical Reagent Co., LTD, China) boil and cool at room temperature. The sections are placed in 3% hydrogen peroxide to blocking endogenous peroxidase activity. Sections were incubated with 50ul diluted p-mTOR, beclin1 antibodies (abcam) at 4°C overnight, then incubated with the corresponding secondary antibody (Servicebio) for 15 min, and then perform DAB chromogenic reaction (Servicebio). Visualize staining of tissue under a microscope, acquisitive and analysis image.

**1.2 Transmission Electron Microscopy (TEM)**

The proximal colon samples were collected and immediately transferred to glutaraldehyde (2.5%, China National Pharmaceutical Group Chemical Reagent Co., LTD) at 4°C for 4h, postfixed with 1% OsO_4_ (China National Pharmaceutical Group Chemical Reagent Co., LTD) and dehydrated in graded alcohol concentrations (30%, 50%, 70%, 80%, 90% and 95%) for about 15min at each step, then dehydrated by a graded series of acetone (90%, 95%) for about 15min at each step. The specimen was then embedded in Spurr resin (SPI-CHEM). The specimen was sectioned using the LEICA EM UC7 ultratome (Leica Microsystems GmbH, Wetzlar, Germany), and sections were stained with uranyl acetate (SPI-CHEM) and alkaline lead citrate (China National Pharmaceutical Group Chemical Reagent Co., LTD). These sections were observed by TEM (H-7650; Hitachi, Tokyo, Japan).

## Supplementary Tables

**Table. S1. PCR primers used for the RT-PCR** **analyses.**

| **Gene** | **Forward Sequence (5'-3')** | **Reverse Sequence (5'-3')** |
| --- | --- | --- |
| **β-actin** | **AGTGTGACGTTGACATCCGT** | **GCAGCTCAGTAACAGTCCGC** |
| **CB1** | **CTGATGTTCTGGATCGGAGTC** | **TCTGAGGTGTGAATGATGATGC** |
| **CB2** | **TGACAAATGACACCCAGTCTTCT** | **ACTGCTCAGGATCATGTACTCCTT** |
| **Occludin** | **TTCCTCTGACCTTGAGTGTGG** | **CTCTTGCCCTTTCCTGCTTT** |
| **Claudin-1** | **TGCCCCAGTGGAAGATTTACT** | **CTTTGCGAAACGCAGGACAT** |
| **ZO-1** | **GCCGCTAAGAGCACAGCAA** | **GCCCTCCTTTTAACACATCAGA** |
| **mTOR** | **AGAAGGGTCTCCAAGGACGACT** | **GCAGGACACAAAGGCAGCATTG** |
| **beclin-1** | **CAGCCTCTGAAACTGGACACGA** | **CTCTCCTGAGTTAGCCTCTTCC** |
| **LC3-II** | **GTCCTGGACAAGACCAAGTTCC** | **CCATTCACCAGGAGGAAGAAGG** |
| **Atg5** | **CTTGCATCAAGTTCAGCTCTTCC** | **AAGTGAGCCTCAACCGCATCCT** |
| **Atg7** | **CCTGTGAGCTTGGATCAAAGGC** | **GAGCAAGGAGACCAGAACAGTG** |
| **Atg9a** | **GTTAGCTGTGGAACACGTCCTC** | **GCAAGAATCACTCGGAGCAGCT** |
| **Atg12** | **GAAGGCTGTAGGAGACACTCCT** | **GGAAGGGGCAAAGGACTGATTC** |
| **TLR4** | **ATGGCATGGCTTACACCACC** | **GAGGCCAATTTTGTCTCCACA** |
| **CD14** | **ACTTCTCAGATCCGAAGCCAG** | **CCGCCGTACAATTCCACAT** |
| **MyD88** | **AGGACAAACGCCGGAACTTTT** | **GCCGATAGTCTGTCTGTTCTAGT** |

**Abbreviation: CB1, cannabinoid receptor 1; CB2, cannabinoid receptor 2.**

**Table S2. Reagents.**

| **Reagent** | **Source** | **Identifier** |
| --- | --- | --- |
| **p-mTOR** | **abcam** | **ab109268** |
| **beclin1** | **abcam** | **ab210498** |
| **ZO-1** | **Servicebio** | **GB111402** |
| **occludin** | **Servicebio** | **GB111401** |
| **claudin-1** | **Servicebio** | **GB11032** |
| **Glycocholic Acid-[2H4]/D4-GCA** | **IsoSciences** | **13443** |
| **Taurocholic Acid-[D4] Na Salt /D4-TCA** | **IsoSciences** | **13232** |
| **Glycodeoxycholic Acid-d4 /D4-GDCA** | **IsoSciences** | **ZIS-13226** |
| **Cholic-2,2,4,4-d4 Acid/D4-CA** | **IsoSciences** | **13098** |
| **Deoxycholic Acid-d4/D4-DCA** | **IsoSciences** | **ZIS-13100** |
| **Lithocholic-2,2,4,4-d4 Acid/D4-LCA** | **IsoSciences** | **13099** |
| **Ethyl acetate** | ***China National Pharmaceutical Group Chemical Reagent Co., LTD*** | **10009418** |

**3 Supplementary Figures**

**Supplementary Figure S1. *A. muciniphila* reduced colon epithelial injury.** (A) Representative images of ZO-1, occludin, and claudin-1 immunofluorescence staining (left panel). Relative colon mRNA expression of ZO-1, occludin, and claudin-1 among three groups (right panel). (B) Representative AB-PAS stained images of the colon. **P* < 0.05, ***P* < 0.01, ****P* < 0.001.
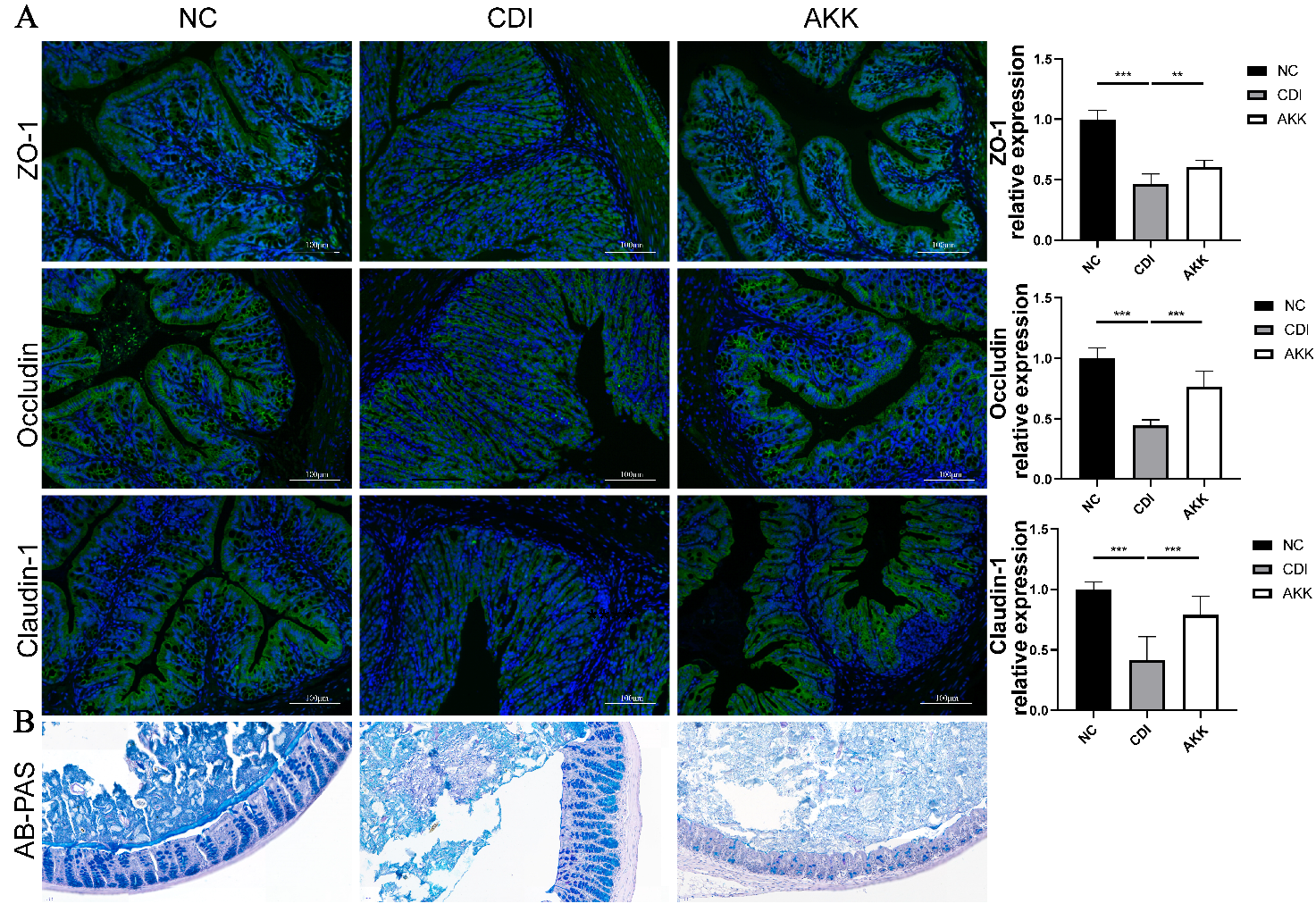


**Supplementary Figure S2.** **CDI mice gut microbial community structure.** (A) *A. muciniphila*load in feces was determined on day 6 after infection. (B) The bacterial taxa of the NC group (red) were compared with those of the CDI group (blue) at different levels (LDA score > 4). ****P* < 0.001.


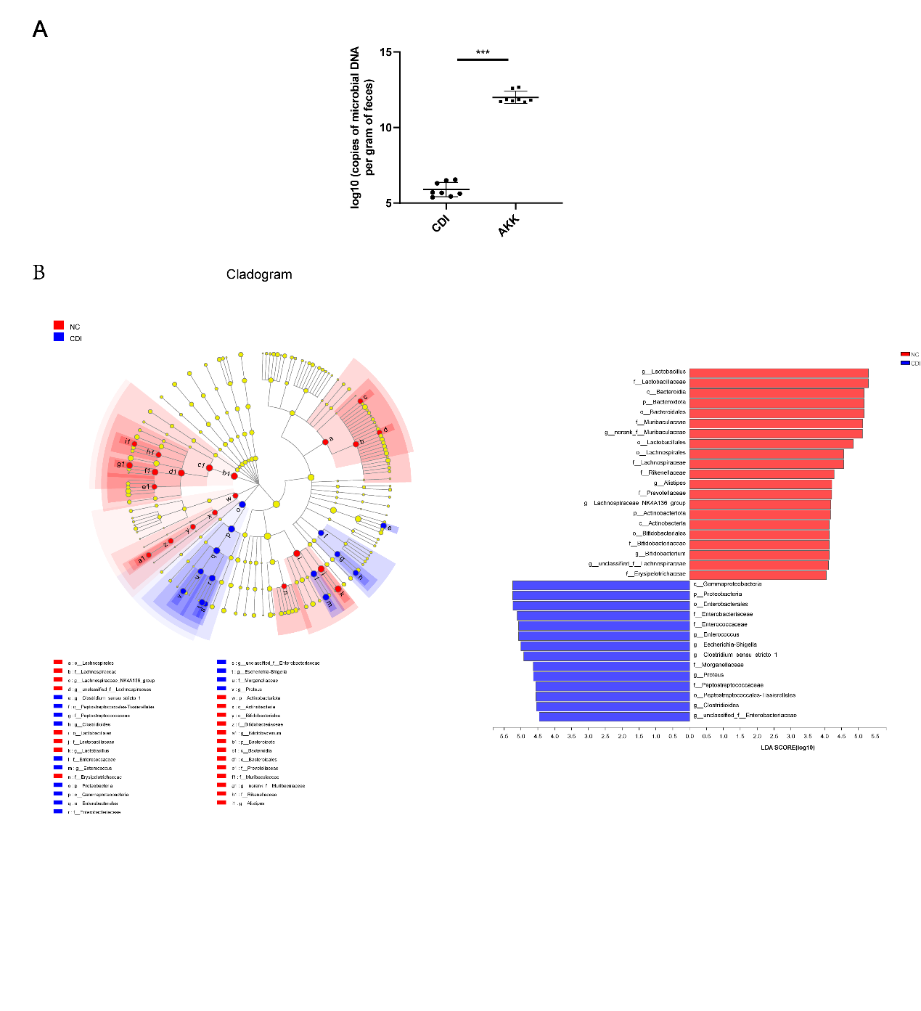


**
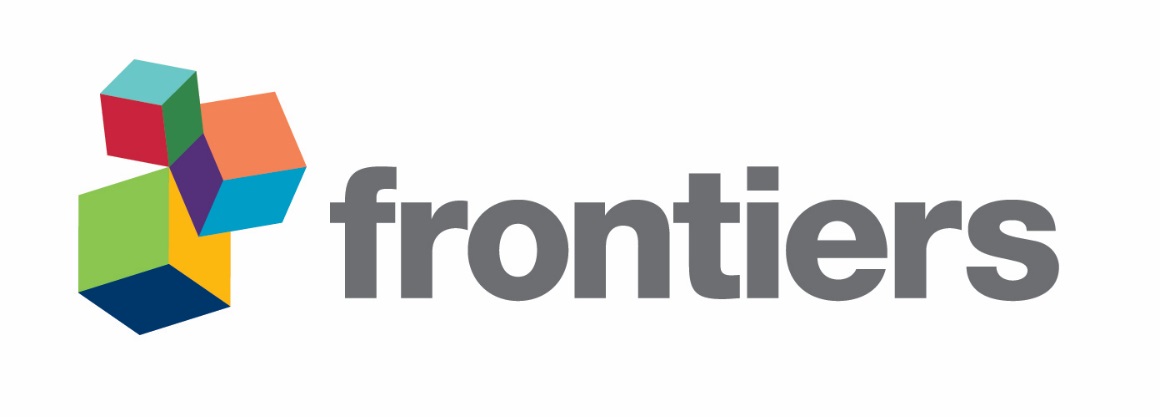
**
